# Exogenous melatonin activates the antioxidant system and maintains postharvest organoleptic quality in Hami melon (*Cucumis. melo* var. *inodorus* Jacq.)

**DOI:** 10.3389/fpls.2023.1274939

**Published:** 2023-10-30

**Authors:** Yue Wang, Minrui Guo, Weida Zhang, Yujie Gao, Xiaoqin Ma, Shaobo Cheng, Guogang Chen

**Affiliations:** ^1^ College of Food Science and Technology, Shihezi University, Shihezi, Xinjiang, China; ^2^ Research Center of Xinjiang Characteristic Fruit and Vegetable Storage and Processing Engineering, Ministry of Education, Shihezi, Xinjiang, China

**Keywords:** melatonin, Hami melon, antioxidant capacity, postharvest storage, reactive oxygen species, fruit quality

## Abstract

Hami melon is prone to postharvest perishing. Melatonin is a signaling molecule involved in a variety of physiological processes in fruit, and it improves fruit quality. We hypothesized that melatonin treatment would improve the storage quality of Hami melon by altering its respiration and reactive oxygen species (Graphical abstract). Our results indicated that optimal melatonin treatment (0.5 mmol L^−1^) effectively slowed the softening, weight loss, and respiratory rate of the Hami melon fruit. Furthermore, melatonin markedly improved the antioxidant capacity of the fruit and protected it from oxidative damage by decreasing its contents of superoxide anions, hydrogen peroxide, and malondialdehyde. Melatonin significantly enhanced the activities of superoxide dismutase, catalase, ascorbate peroxidase, and peroxidase. The total phenol, total flavonoids, and ascorbic acid contents were maintained by melatonin treatment. This treatment also repressed the activities of lipase, lipoxygenase, and phospholipase D, which are related to lipid metabolism. Thus, exogenous melatonin can maintain postharvest organoleptic quality of Hami melon fruit by increasing its antioxidant activity and inhibiting reactive oxygen species production.

## Graphical Abstract

After melatonin treatment, the respiratory rate and ROS accumulation of Hami melon were inhibited. Lower respiratory rate can reduce nutrient loss, and maintain higher levels of TSS and TA, which helps delay the loss in fruit firmness and weight. In addition, ROS accumulation can activate the antioxidant system, leading to an increase in antioxidant enzymes and substances. This helps to enhance the antioxidant capacity of the fruit, which in turn acts on ROS and inhibits accumulation of ROS. Excessive ROS can accelerate membrane lipid peroxidation and MDA production, leading to membrane lipid damage and fruit senescence. MT treatment maintained the cell membrane integrity and delayed fruit senescence. Notes: EX-MT, exogenous melatonin; ROS, reactive oxygen species; TSS, total soluble solid content; TA, titratable acid content; SOD, superoxide dismutase; POD, peroxidase; CAT, catalase; APX, ascorbate peroxidase; AsA, ascorbic acid; TPC, total phenols content; TFC, total flavonoids content; MDA, malondialdehyde; DPPH, 1,1-diphenyl-2-picrylhydrazyl; ABTS, 3-ethylbenzthiazoline-6-sulfonic acid.

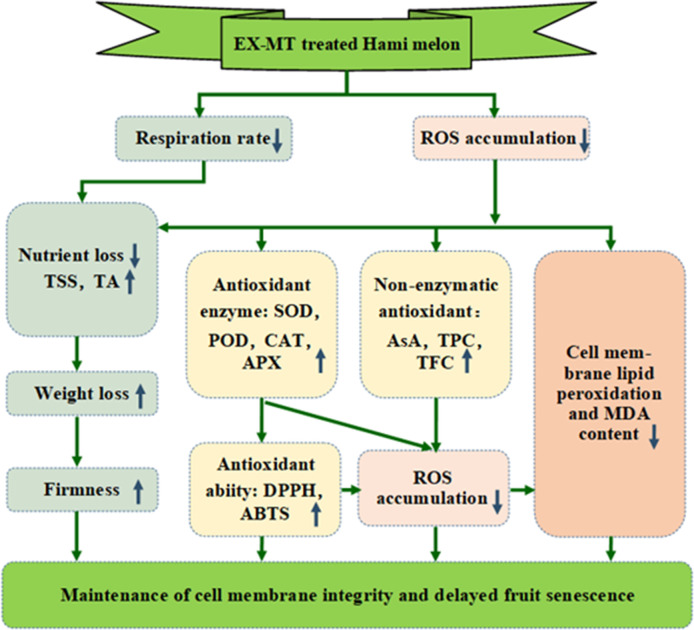

## Highlights

- Melatonin (MT) delays postharvest senescence and maintains fruit quality
- MT decreases respiratory rate and nutrient loss
- MT increases total phenolic and flavonoid contents, as well as antioxidant capacity
- MT decreases ROS accumulation and increases antioxidant enzyme activities
- MT inhibits membrane lipid peroxidation

## 1 Introduction

Hami melon (*Cucumis. melo* var. *inodorus* Jacq.) is widely produced in Xinjiang, China and enjoyed by consumers owing to its unique sweet and sour taste, and nutrient quality (Zhang et al., 2017a). Its rapid postharvest senescence makes it difficult to store and transport thereby decreasing its sensory quality and commercial value and resulting in huge economic losses. Thus, methods for mitigating fruit decay and maintaining their antioxidant potential are required. Cold storage is used extensively for prolonging the postharvest life of fruit. However, Hami melon is prone to cold damage under cold storage, so it is not suitable for delaying postharvest senescence and improving antioxidant capacity. Therefore, in previous studies safe and ecofriendly postharvest approaches, such as the application of 1-MCP (Li et al., 2011), sodium silicate (Bi et al., 2006), nitric oxide (Zhang et al., 2017b), and oxalic acid (Wang et al., 2018), have been investigated.

Melatonin (MT), a natural indole compound, was identified in higher plants in 1995 (Dubbels et al., 1995; Wang et al., 2020b). As an endogenous bioactive and antioxidative molecule, MT positively influences ripening (Dong et al., 2021), free-radical scavenging (Lin et al., 2019), and senescence (Deng et al., 2021). Several studies have investigated MT treatment as a means to prolong the shelf life of fruits (Ze et al., 2021). For instance, Onik et al. (2021) found that 1 mmol L^–1^ MT treatment reduces ethylene production and increases the activity of antioxidant enzymes in apples. MT treatment (0.5 mmol L^–1^) improves the antioxidant activity and delays senescence in blueberries (Shang et al., 2021). Dong et al. (2021) reported that MT treatment delays ripening of mango fruit during storage. However, there have been no reported studies on how MT treatment affects the respiration, antioxidant system, and postharvest organoleptic quality of Hami melon fruit.

Postharvest senescence of fruit is related to excessive accumulation of reactive oxygen species (ROS) (Gao et al., 2016). During postharvest storage, excessive ROS damage cell membranes, while low levels of ROS are scavenged by the antioxidant system (Meitha et al., 2020). The antioxidant system comprises enzymatic and non-enzymatic factors. The former primarily involves superoxide dismutase (SOD), catalase (CAT), ascorbate peroxidase (APX), and peroxidase (POD) (Zhang et al., 2020), and the latter involves ascorbic acid (AsA), phenols (measured as total phenol content; TPC), and flavonoids (measured as total flavonoid content; TFC) (Yang et al., 2021). Both systems play important roles in preventing oxidative damage to fruit. With fruit senescence, its antioxidant capacity decreases, leading to ROS accumulation, increased phospholipase D (PLD) and lipoxygenase (LOX) activities, and increased malondialdehyde (MDA) content, all of which lead to membrane lipid peroxidation (Dong et al., 2021).

This research aimed to investigate the physio-biochemical changes in response to MT in Hami melon fruit. The specific parameters and metabolic processes analyzed in this study included the physiological index and ROS generation, membrane lipid metabolism, and the antioxidant system. The mechanisms underlying the regulatory role by which MT inhibits senescence of Hami melon fruit were discussed.

## 2 Materials and methods

### 2.1 Experimentation

**Figure 1:**
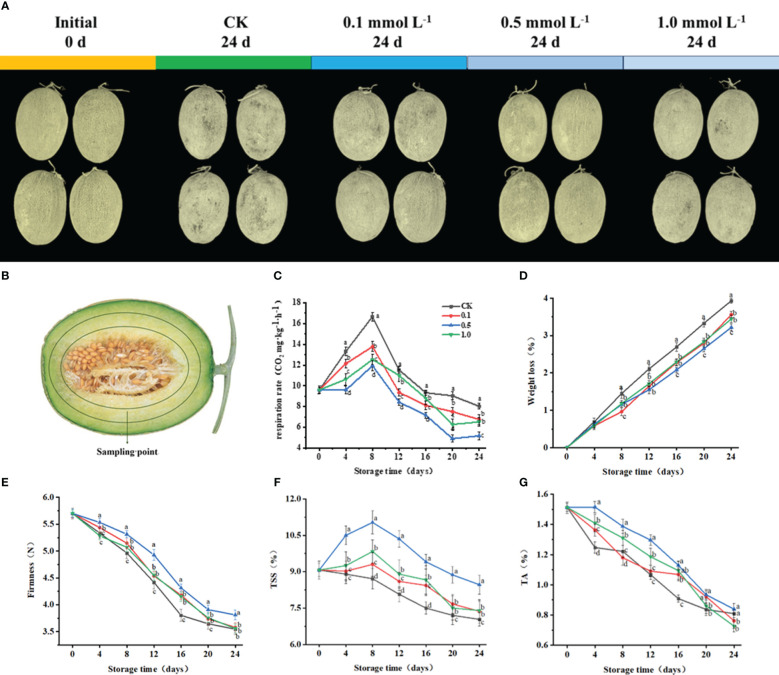
Visual appearance of Hami melon fruit treated with 0,0.1, 0.5, 1.0 mmol L-1 MT for 24 d **(A)**, Sampling point of Hami melon fruit **(B)** and changes of melatonin treatments on the quality of Hami melon fruit stored at 6 ± 1 °C. Notes: **(C)** Respiration rate; **(D)** Weight loss; **(E)** Firmness; **(F)** TSS, total soluble solid content; **(G)** TA, titratable acid content. A total of 228 Hami melons were divided into four groups (n=57/group). All values are means ± SE. Error bars represent the standard error of the means. Values followed by different superscripts (a-d) are significantly different in the same sampling time (*p*< 0.05).

Hami melons (*N* = 228; variety ‘Xizhoumi 17’; total soluble solid content (TSS) 9.0%; average weight 1.8 kg) free from mechanical damage and of similar size and shape, were purchased from local orchards in Hami, Xinjiang, China. They were divided into four treatment groups (57 per group), soaked in MT solutions with concentrations of 0 (control), 0.1, 0.5, or 1.0 mmol L^−1^ for 10 min (**Figure 1A**), air-dried, and stored at 6 ± 1°C (preliminary test results showed that chilling injury occurred at storage temperatures lower than 5°C). On days 0, 4, 8, 12, 16, 20, and 24, the respiration rate, pulp firmness, TSS, titratable acid (TA), and AsA content were determined. Furthermore, for the rigor of the experiment, fruit pulp (1.5 cm from the peel) was collected (**Figure 1B**), immediately frozen in liquid nitrogen, and stored at −80°C.

### 2.2 Measurement of fruit quality parameters

The respiration rate was evaluated as previously described by Yang et al. (2021). Three Hami melons were selected from each group and placed in a 37-L sealed container at 22 ± 2°C for 2 h. Then, the headspace gas (1 mL) was collected for gas chromatographic analysis (GC-16A, Shimadzu, Japan). Respiration rate is expressed as CO_2_ released per kilogram per hour of fresh weight (mg kg^−1^ h^−1^).

Weight loss (%) was assessed with 15 fruit per group based on the following formula.


$$\text{Weight loss }\left(\%\right)=\frac{Initial\;weight-Final\;weight}{Initial\;weight}\times 100\text{ \%}.$$

Three points at the pulp of each Hami melon were selected to measure fruit firmness using a hardness tester (GY-4, Zhejiang Yueqing Aidebao Instrument Co, Ltd., China). The unit of fruit firmness is Newtons (N).

Fresh Hami melon pulp (5 g) was ground and filtered. TSS content in the filtrate was measured with a GMK-701R digital refractometer (G-Won Hitech, Seoul, Korea). TA content was determined using the method of Chater et al. (2018). TA and TSS are expressed as %.

### 2.3 Measurement of O_2_^−^ production rate and H_2_O_2_ content

The O_2_^−^ production rate was determined by the methods of Yang et al. (2021). A 5 g quantity of sample was ground in 5 mL of phosphate buffer and centrifuged for 15 min (4°C, 12,000 × g). Then, 1 mL of phosphate buffer and 1 mL of hydroxylamine chloride reagent were added to 1 mL of the supernatant mix and left at 25°C for 1 h. A total of 0.5 mL of the incubation solution was mixed with 1 mL of 17 mmol L^−1^ p-aminobenzene sulfonic acid and 1 mL of 7 mmol L^−1^ alpha-naphthylamine. This reaction mixture was left at 25°C for 20 min and the absorbance was read at 530 nm. The result was expressed as μmol kg^−1^ s^−1^.

The H_2_O_2_ content was determined according to the method of Zhang et al. (2022). The results are expressed as mmol kg^−1^.

### 2.4 Measurement of LOX and PLD activities and MDA content

LOX activity (U kg^−1^) was evaluated according to the method described by Sun et al. (2022). Extraction of crude enzyme solution: 5.0 g of pulp tissue was placed in a mortar, ground with liquid nitrogen, added to 5 mL phosphate buffer precooled at 4°C, centrifuged at 12,000 × g (4°C) for 30 min, and the supernatant was used for LOX activity determination. Phosphate buffer (2.7 mL, pH = 6.8, 50 mmol L^−1^) containing 100 μL 0.5% linoleic acid solution was held at 30°C for 10 min, and then mixed well with 200 μL crude enzyme solution. It was determined at 234 nm and expressed in units of 0.001 increase in absorbance per hour.

PLD activity was measured by the choline Reinecke salt precipitation method described by Sharafi et al. (2021), and the results (U kg^−1^) are expressed as a change of 0.1 in absorbance at 520 nm per hour.

The thiobarbituric acid colorimetry method was used to measure MDA content (Wei et al., 2022). Briefly, a total of 1.0 g of pulp sample was homogenized with 5 mL of 5% trichloroacetic acid (TCA) solution, and then centrifuged at 10,000 × g for 20 min at 4°C. Then, 2 mL of the supernatant was mixed with 2 mL of 0.67% thiobarbituric acid and heated for 20 min in a water bath at 100°C, then centrifuged again (10 min) after cooling. The absorbance values of the supernatant at 450, 532, and 600 nm were determined, respectively. The results are expressed as μmol kg^−1^.

### 2.5 Measurement of antioxidant enzymatic activity

A modified guaiacol method was used to measure POD activity (Yang et al., 2021). The reaction mixtures contained 3 mL of 25 mmol L^−1^ guaiacol solution and 0.5 mL of enzyme extract. After 200 μL of 0.5 mol L^−1^ H_2_O_2_ solution was added and mixed quickly, a timer was started. The initial absorbance value at 470 nm was recorded after 15 s. Data were collected every 60 s for 6 min. POD activity is expressed as a change of 1 in absorbance per minute per gram of fresh melon. The results are represented as U kg^−1^.

CAT activity was measured using Solarbio (Beijing, China) CAT kits according to the manufacturer’s instructions. The results are defined as the degradation of 1 mol H_2_O_2_ per minute per gram of fresh pulp in the reaction system and are represented as U kg^−1^.

SOD activity was measured according to the method of Sun et al. (2022) at maximum absorption (560 nm). One unit of SOD activity is defined as the amount of enzyme that inhibits the photoreduction of nitro blue tetrazolium chloride by 50% and is represented as U kg^−1^.

APX activity was evaluated using the method described by Sun et al. (2022). Fruit pulp (5 g) was used for the preparation of crude enzyme extracts, and then 2.6 mL reaction buffer, 0.1 mL enzyme extract, and 0.3 mL 2 mmol L^−1^ H_2_O_2_ were added. After 15 s, the initial value was measured at 290 nm. Data were collected every 30 s for 3 min. APX activity is defined as a change of 0.01 in absorbance per minute per gram of fresh melon and is represented as U kg^−1^.

### 2.6 Measurement of non-enzymatic antioxidant and antioxidant capacity

Fruit pulp (0.1 g) was added to 5 mL of 80% methanol. Then, the mixture was ground, homogenized, and centrifuged at 11,000 *×* g for 30 min at 4°C. The supernatant was used to determine TPC and TFC.

TPC was evaluated by the Folin–Ciocalteu colorimetric method (Rastegar et al., 2020) with gallic acid as the standard. The absorbance for TPC was determined at 280 nm. TFC was evaluated using the method described by Wei et al. (2022). A standard quercetin calibration curve was used to estimate TFC. The absorbance for TFC was determined at 325 nm. AsA content was evaluated according to Fan et al. (2018). The results of TPC, TFC, and AsA are expressed as g kg^−1^.

The antioxidant capacity was evaluated by measuring 1,1-diphenyl-2-picrylhydrazyl (DPPH)- and 3-ethylbenzthiazoline-6-sulfonic acid (ABTS)-radical-scavenging capacities according to the methods of Rastegar et al. (2020). Results are expressed as %.

### 2.7 Statistical analysis

All the experimental data were statistically analyzed using SPSS 23 (SPSS Inc., Chicago, IL, USA). The results are presented as mean ± standard error of three replicates. Principal component analysis (PCA) and Pearson’s correlation analysis were conducted using SPSS 23. Statistical differences between samples were determined using Duncan’s multiple range test. *p*< 0.05 was considered significant.

## 3 Results

### 3.1 Changes in Hami melon fruit quality

To reflect the quality of the fruit, this study measured respiratory rate, weight loss, TSS, TA, and firmness. The control and MT-treated groups (**Figure 1C**), all reached their peak respiratory rate at 8 d, with values of 16.7 (control), 13.8 (0.1 MT group), 11.9 (0.5 MT group) and 12.5 mg kg^−1^ h^−1^ (1.0 MT group), respectively. The 0.5 MT group shows a 28.4% lower respiratory rate than the control group at 8 d.

Throughout storage, the weight losses of the MT and control groups show an increasing trend (**Figure 1D**). At 24 d, the weight loss of the control group is 3.9%, while that of the 0.5 MT group is the lowest (3.2%). The weight loss for the 0.5 MT group is 22.2% lower than that of the control group at 24 d.

Firmness for all groups gradually decreases with storage (**Figure 1E**), with that of the 0.5 MT group being 7.5% higher than that of the control group at 24 d.

TSS in the MT-treated groups peak at 8 d with values of 9.3% (0.1 MT group), 11.03% (0.5 MT group), and 9.8% (1.0 MT group) (**Figure 1F**). TSS in control and 0.5 MT group decrease on days 8–24 and reach their lowest values (7.0% and 8.5%, respectively) at 24 d.

TA content gradually decreases for all groups during storage (**Figure 1G**). The MT groups show higher TA contents than that of the control group. TA content for the 0.5 MT group is 3.7% higher than that of the control group at 24 d.

### 3.2 Changes in ROS level

**Figure 2:**
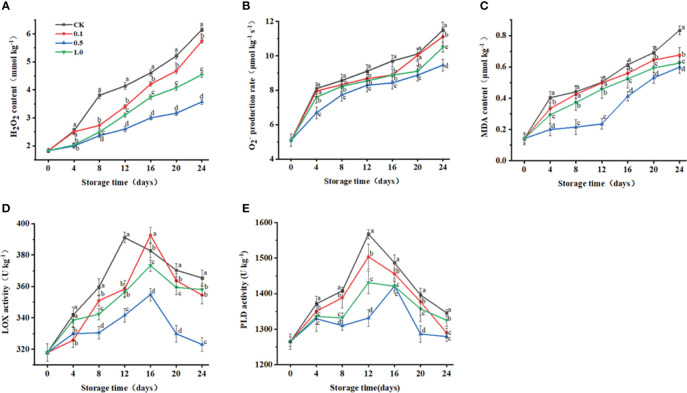
Changes of melatonin treatments on H_2_O_2_ content **(A)**, O_2_^-^ production rate **(B)**, MDA content **(C)**, and LOX **(D)** and PLD **(E)** activities of Hami melon (57 per group) during storage at 6 ± 1°C. Notes: MDA, malondialdehyde; LOX, lipoxygenase; PLD, phospholipase **(D)** A total of 228 Hami melons were divided into four groups (n=57/group). All values are means ± standard error. Error bars represent the standard error of the means. Values followed by different superscripts (a-d) are significantly different in the same sampling time (*p*< 0.05).

Since oxidative stress can cause fruit senescence, we evaluate the cellular oxidative state by measuring ROS levels (Yang et al., 2021). Continuous increases in O_2_^−^ production rate and H_2_O_2_ content are observed in all groups as storage proceeds, while the latter exhibits a lower O_2_^−^ production rate and H_2_O_2_ content than the former at all-time points (**Figures 2A, B**). The O_2_^−^ production rate and H_2_O_2_ content for the 0.5 MT group are 17.7% and 42.0% lower than those for the control group at 24 d.

### 3.3 Changes in MDA content, PLD activity, and LOX activity

MDA content is an important indicator of the degree of peroxidation in plant cells (Yang et al., 2021). In this study, MDA content for the control and MT groups gradually increases to a maximum at the end of storage (**Figure 2C**), while the 0.5 MT group shows the lowest value (0.6 μmol kg^−1^) at 24 d.

The degradation of structural membrane lipids is the main cause of cell membrane loss, and PLD and LOX are two major degrading enzymes participating in this process (Zhang et al., 2022). LOX activities reach their peaks at 12 d (control, 391.1 U kg^−1^), 16 d (0.1 MT, 392.6 U kg^−1^), 16 d (0.5 MT, 354.8 U kg^−1^), and 16 d (1.0 MT, 373.2 U kg^−1^), and then decrease during the remaining days (**Figure 2D**). LOX activity for the 0.5 MT group is lower than that for the control group throughout storage.

MT treatment (0.5 mmol L^−1^) delays the PLD peak to the 16th day, while those of the other groups peak at 12 d (**Figure 2E**). PLD activity for the 0.5 MT group is lower than that for the control group throughout storage.

### 3.4 Changes in enzymatic antioxidant activity

**Figure 3:**
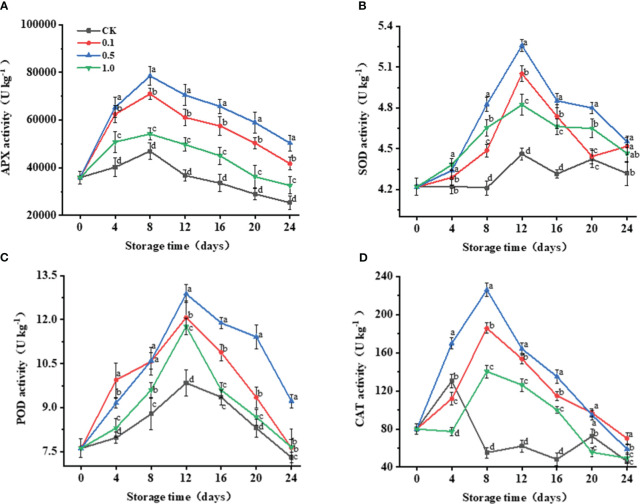
Changes of melatonin treatments on antioxidant enzyme activities of Hami melon fruit (57 per group) during storage at 6 ± 1°C. **(A)** APX, ascorbate peroxidase; **(B)** SOD, superoxide dismutase; **(C)** POD, peroxidase; **(D)** CAT, catalase. A total of 228 Hami melons were divided into four groups (n=57/group). All values are means ± standard error. Error bars represent the standard error of the means. Values followed by different superscripts (a-d) are significantly different in the same sampling time (*p*< 0.05).

Key antioxidant enzymes such as APX, CAT, SOD, and POD, usually play an important role in maintaining the balance of ROS (Zhang et al., 2020). APX activity for the control and MT-treated groups reach their peaks at 8 d (**Figure 3A**), with that for the 0.5 MT group being 98.3% higher than that of the control group.

POD and SOD activities for the control and MT groups exhibit similar trends (**Figures 3B, C**), continuously increasing from 0 to 12 d and dropping thereafter. The 0.5 MT group shows the highest POD and SOD activities, which are 30.8% and 17.8% higher than those of the control group at 12 d, respectively.

For the control group, CAT activity peaks on day 4, while those in the MT groups peak on day 8 (**Figure 3D**). The peak CAT activity of the control group only increases by 63.1% compared with its initial value, while that of the 0.5 MT group increases by 182.2%. 3.5 Changes in non-enzymatic antioxidant capacity.

**Figure 4:**
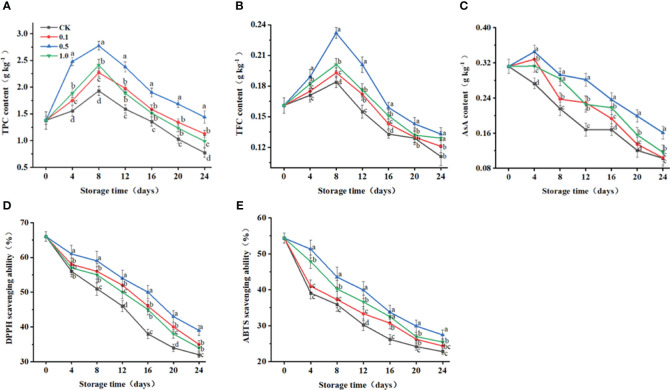
Changes of melatonin treatments on non-enzymatic and enzymatic antioxidant capacity of Hami melon (57 per group) during storage at 6 ± 1°C. **(A)** TPC, total phenols content; **(B)** TFC, total flavonoids content; **(C)** ASA, ascorbic acid; **(D)** DPPH radical scavenging activity, 1,1-diphenyl-2-picrylhydrazyl; **(E)** ABTS radical scavenging activity, 3-ethylbenzthiazoline-6-sulfonic acid. A total of 228 Hami melons were divided into four groups (n=57/group). All values are means ± standard error. Error bars represent the standard error of the means. Values followed by different superscripts (a-d) are significantly different in the same sampling time (*p*< 0.05).

Exogenous melatonin can influence secondary of Hami melon fruit. TPC and TFC reach their peaks at 8 d and then decrease during days 8-24 (**Figures 4A, B**). TPC and TFC for the MT groups are consistently higher than those for the control group. Specifically, at 8 d, TPC and TFC for the 0.5 MT group are 43.7% and 26.1% higher than those for the control group.

Unlike the continuously decreasing trend for the control group, AsA contents for the MT groups first increase during days 0–4 and then decrease for days 4–24 (**Figure 4C**). At 24 d, the AsA content in the control group is decreased by 67.0%, while those for the 0.1, 0.5 and 1.0 MT groups are decreased by 66.7%, 48.4%, and 62.7%, respectively.

DPPH- and ABTS-radical-scavenging capacities gradually decrease for all groups during storage (**Figures 4D, E**). At 24 d, DPPH- and ABTS-radical-scavenging capacities for the 0.5 MT group are 85.9% and 20.3% higher than those for the control group, respectively.

### 3.6 PCA and Pearson correlation

**Figure 5:**
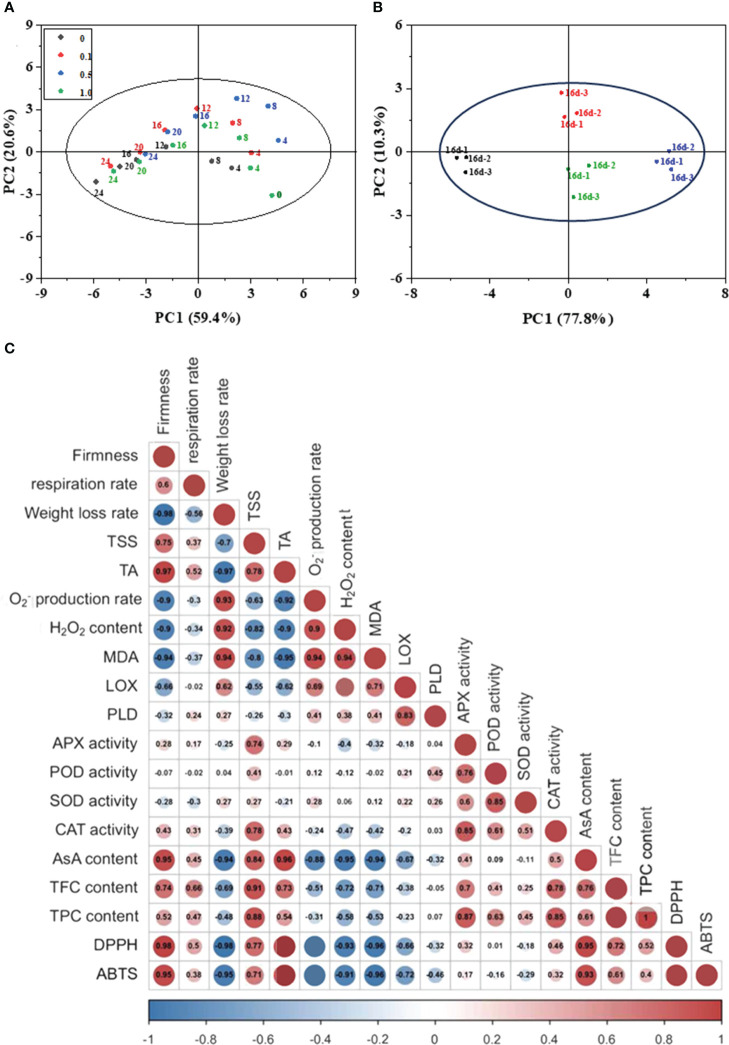
**(A)** Principal component analysis (PCA) of the parameters of Hami melon stored at 6 ± 1°C for 24 d; **(B)** Principal component analysis (PCA) of the parameters of Hami melon after 16 d storage; **(C)** Correlation analysis of melatonin treatments and Hami melon fruit quality parameters and antioxidant system during storage (24 d). Correlation coefficient is proportional to numerical value and color intensity; three repetitions per treatment.

PCA was used to determine the principal components responsible for the overall variance in all analyzed traits (59.4% and 20.6% for PC1 and PC2; **Figure 5A**). The results demonstrate that the effect of storage time is more significant than MT treatment on the intrinsic quality and antioxidant capacity of Hami melon. There are obvious separations between the control and 0.5 MT groups. An insignificant separation is observed between the 0.1 and 1.0 MT groups. On day 16, PCA generated two components, PC1 (TSS, O_2_^−^ production rate, LOX, CAT, and DPPH) and PC2 (PLD, MDA, TFC, and POD; **Figure 5B**). A significant difference between the control and 0.5 MT group in the direction of PC1 are observed. These findings suggest that the quality and antioxidant activity of Hami melon show discrepancies among different MT and control groups.

Pearson matrix analysis highlights the intercorrelations between various indicators. The correlation analysis results (**Figure 5C**) indicate that MDA content is positively correlated with H_2_O_2_ content (*r* = 0.94) and O_2_^−^ production rate (*r* = 0.94), and that LOX activity is positively correlated with PLD activity (*r* = 0.83) and MDA content (*r* = 0.71). These results indicate that with increasing ROS levels and LOX and PLD activity, lipid peroxidation in Hami melon is aggravated, eventually leading to oxidative membrane damage and increased MDA content.

APX activity is positively correlated with POD (*r* = 0.76), SOD (*r* = 0.60), and CAT (*r* = 0.85) activity. In Hami melon, the combined action of O_2_^−^-scavenging SOD and H_2_O_2_-metabolizing APX, POD, and CAT is responsible for oxidant-resistance during storage. Furthermore, AsA content, TPC, and TFC are negatively correlated with O_2_^−^ production rate and H_2_O_2_ content. AsA content shows a significant correlation with DPPH- (*r* = 0.95) and ABTS-radical scavenging activities (*r* = 0.93).

## 4 Discussion

Hami melon readily deteriorates during postharvest storage by juice exudation, fruit softening, and nutrient loss, which seriously affects its nutritional quality and commercial value (Zhang et al., 2022). Studies have confirmed that MT treatment can increase antioxidant capacity and slow senescence in blueberries, mangoes, peaches, and bananas (Gao et al., 2016; Dong et al., 2021; Shang et al., 2021; Wang et al., 2022). Therefore, as a safe and nontoxic substance, MT shows practical potential for application in postharvest fruit. To explore the mechanism of this action in Hami melon, we analyzed physiological index and ROS generation, membrane lipid metabolism, and the antioxidant system.

Respiration is a physiological process by which fruit activity can be evaluated (Goffi et al., 2020). Fruit firmness reflects the maturity and edible quality of fruit (Ma et al., 2022). Hami melon as a kind of climacteric fruit that shows a respiratory peak during storage. MT treatment at 0.5 mmol L^−1^ represses the reduction in firmness and increase in respiration rate (**Figures 1C, E**). The maintenance of fruit firmness may be due to MT repressing the respiration rate of fruit and retarding the activity of cell wall enzymes, such as polygalacturonase, pectin lyase, pectin methylesterase, and cellulase, as well as starch-degradation-related enzymes (Tang et al., 2020; Wang et al., 2020a), thereby maintaining pulp cell structure and delaying fruit softening. Studies have also confirmed that MT application can markedly preserve firmness in mangoes (Dong et al., 2021), pomegranates (Aghdam et al., 2020), and peaches (Gao et al., 2016), while Fan et al. (2022) showed that MT-treatment maintains firmness and slows respiration in apples.

Soluble sugars and organic acids are not only essential nutritional components and pivotal flavor compounds in fruit, they also play a key role in sweet and sour flavors (Ma et al., 2021; Zhang et al., 2023). During post-ripening and senescence, a higher respiratory rate accelerates the consumption of nutrients, leading to decreases in TSS and TA (Zhao et al., 2020). In the current work, TSS content increased on days 0–8, which may be related to the decomposition of starch into glucose (**Figure 1F**). The MT groups show higher TSS and TA contents, which may be due to MT inhibiting respiration, correspondingly reducing substrate consumption and maintaining TSS and TA contents (**Figure 1G**). Onik et al. (2021) reported that MT treatment (1 mmol L^−1^) effectively reduces the respiration rate and maintains TSS and TA contents in apples. With the senescence of Hami melon fruit, TSS shows a decreasing tendency on days 8–24, which may be due to the excessive consumption of soluble fructose and glucose during glycolysis (Gao et al., 2016).

TA content in Hami melon fruit exhibits a decreasing trend for all groups. This may be for several reasons: Firstly, citric acid, isocitric acid, succinic acid, and other organic acids can be converted to oxaloacetic acid and undergo gluconeogenesis, which produces sugar (glucose or glycogen). Secondly, multiple organic acids directly participate in the tricarboxylic acid cycle, glycolysis, and other respiratory metabolic pathways (Wu et al., 2020). However, during fruit senescence, the degradation rates of organic acids in fruit are higher than their synthesis rates, decreasing their contents (Habibi et al., 2020; Zhang et al., 2021). Thirdly, metallic cations (e.g., Fe^2+^ and Mg^2+^) in plant cells can be neutralized by organic acids, forming strong-base–weak-acid salts, thus decreasing acidity (Yang et al., 2022).

MDA is one of the most important products of membrane lipid peroxidation and can be a useful indicator to judge the degree of membrane damage (Xu et al., 2020; Yang et al., 2021). In this study, loss of membrane integrity and function during storage of Hami melon fruit may be related to excess production of ROS (O_2_^–^ and H_2_O_2_), as well as increased LOX activity and MDA content (Zhang et al., 2022). This may be because PLD catalyzes the hydrolysis of structural phospholipids to produce free linolenic acid for enzymatically peroxidation by LOX or non-enzymatically peroxidation by ROS, thereby forming lipid hydroperoxides. Its spontaneous degradation produces ROS and other free radicals, which can start chain reactions for further peroxidation, leading to MDA accumulation (Sharafi et al., 2021; Wang et al., 2022; Gao et al., 2018; Zhang et al., 2022). Correlation analysis further confirms that the rapid increase of MDA content in Hami melon fruit is related to increases in PLD and LOX activity (**Figure 5**). MT treatment improves ROS scavenging ability, thus slowing the excessive accumulation of ROS, which delays lipid peroxidation of fruit cell membrane.

**Figure 6:**
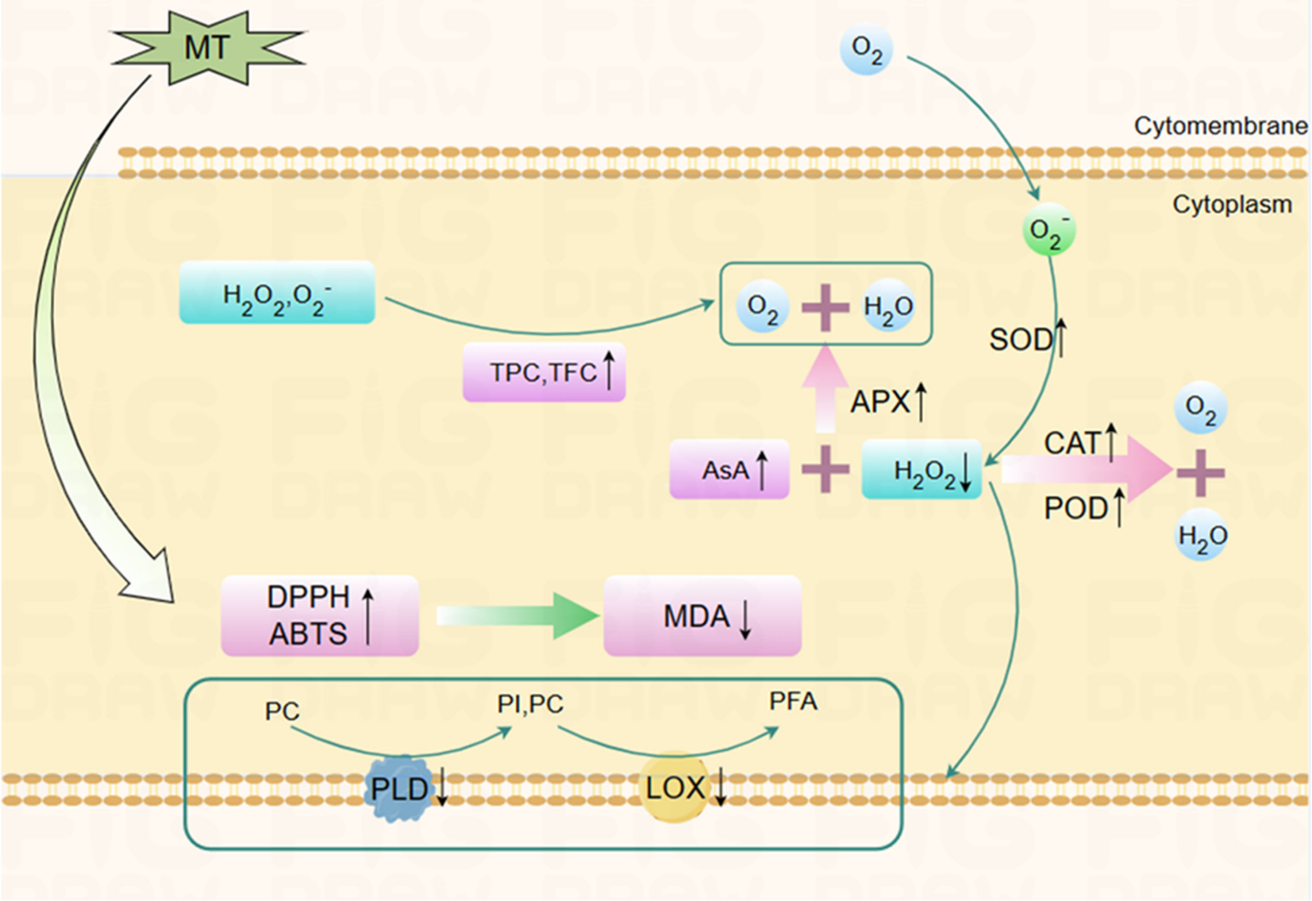
The possible mechanism by which melatonin treatment delays postharvest senescence of Hami melons. After melatonin treatment, SOD, POD, CAT, APX activities, ASA, TPC, TFC content, DPPH, ABTS free radical scavenging ability of Hami melon fruits were enhanced, while PLD, LOX activity, and MDA content were decreased. MT, melatonin; TPC, total phenols content; TFC, total flavonoids content; APX, ascorbate peroxidase; SOD, superoxide dismutase; POD, peroxidase; CAT, catalase; AsA, ascorbic acid; DPPH, 1,1-diphenyl-2-picrylhydrazyl; ABTS, 3-ethylbenzthiazoline-6-sulfonic acid; LOX, lipoxygenase; PLD, phospholipase D; MDA, malondialdehyde; PC, phosphatidylcholine; PI, phosphatidylinositol; PFA, phospholipid fatty acid.

The antioxidant system comprises enzymatic (SOD, POD, CAT, APX) and non-enzymatic systems (AsA, TPC, TFC), etc. (Wang et al., 2022). The enzymatic antioxidant system is mainly responsible for controlling the production of ROS, thus modulating the level of lipid peroxidation to some extent (Shang et al., 2021). SOD, CAT, POD, and APX are vital antioxidant enzymes for eliminating ROS (**Figure 6**). Among them, SOD can specifically dismutate O_2_^−^ into H_2_O_2_ and O_2_ (Gao et al., 2016; Zhang et al., 2022). APX and CAT synergistically convert H_2_O_2_ into H_2_O and O_2_ (Ahmad and Tahir, 2016). POD can catalyze the oxidation of various reducing agents using H_2_O_2_, as represented by RH_2_ + H_2_O_2_ → 2H_2_O + R. Enhanced activities of these antioxidant enzymes and their synergies have been confirmed to be involved in the mechanisms by which lipid peroxidation and senescence are delayed in many horticulture crops. For example, the nitric-oxide-induced senescence delay of Hami melon fruit is recognized as being the result of simultaneously increased antioxidant enzyme activities (Zhang et al., 2017b). Wang et al. (2018) showed that oxalic acid improves SOD, POD, and CAT activities in Hami melon fruit, promoting ROS scavenging and alleviating oxidative damage, thereby delaying senescence.

The present work shows that MT treatment simultaneously increases SOD, POD, CAT, and APX activities, correspondingly decreasing O_2_^−^ and H_2_O_2_ levels (**Figure 3**). Our results are consistent with Fan et al. (2022), who reported that the delay of anthracnose-induced senescence in guava fruit treated with MT may be related to reduced ROS levels, increased SOD, CAT, APX, phenylalanine ammonia lyase activities, and maintenance of TPC and AsA. Correlation analysis further confirmed that the increases of APX, POD, and CAT are related to the decrease of ROS content (**Figure 5**). MT treatment improves antioxidant enzyme activity and maintains the balance of cellular homeostasis. The activation of antioxidant enzymes including SOD and APX by MT treatment has been attributed to the upregulation of antioxidant enzyme mRNA expression in animals, which further indicates that this process might be mediated by MT receptors (Mayo et al., 2002). However, MT receptors have not been identified in plants. Research has shown that MT induces the production of downstream metabolites, i.e., *N*-γ-acetyl-N-2-formyl-5-methoxykynurenamine and cyclic 3-hydroxymelatonin, which are outstanding free-radical scavengers (Ze et al., 2021). Cascades of these compounds can eliminate as many as 10 free radicals, with a 2–4-times better ability to detoxify oxidized molecules than any traditional scavenger (Paredes et al., 2009), which might be why MT treatment is observed to maintain ROS balance in Hami melon fruit in the present study.

Non-enzymatic antioxidants also play vital roles in counteracting the oxidative damage of ROS during senescence. AsA as a major nonenzymatic antioxidant, can directly scavenge ROS (Yang et al., 2022). As demonstrated in this work, MT treatment maintains the AsA content in Hami melon fruit (**Figure 4C**), indicating that non-enzymatic antioxidants might be associated with MT-regulated delay of senescence therein. Higher antioxidant capacity has been linked with enhanced AsA in blueberry fruit treated with MT (Shang et al., 2021). The secondary plant metabolites TPC and TFC in fruit are not only strongly associated with their color development, ripening, aging, and stress resistance, they also play a vital role in their antioxidant potential. In this study, the decrease in TPC of Hami melon fruit observed during late storage may be due to the destruction of ROS by phenolic substances binding to lipid alkoxy radicals, resulting in a decrease in its own content. TPC levels in Hami melon fruit decrease at the later storage stage, which may because flavonoids react with other free radicals through the hydrogen atoms on phenolic hydroxyl groups, forming flavonoid radicals. Correlation analysis further confirmed that AsA, TPC, and TFC are negatively related to O_2_^−^ and H_2_O_2_. In the current study, 0.5 mmol L^−1^ MT treatment increased the TPC and TFC in Hami melon (**Figures 4A, B**), both of which maintained higher levels than those of the control group. Similar findings have also been reported for strawberries and sweet cherries (Liu et al., 2018; Sharafi et al., 2021). Previous findings have also indicated that TPC is precursor of TFC synthesis and that as its content increases, TFC synthesis accelerates (Yang et al., 2021). It may be speculated that enhancement of TFC by MT treatment is associated with enhancement of TPC.

The ABTS method is an effective means by which to measure total antioxidant capacity, and DPPH is a stable nitrogen-centered free radical that is widely used to evaluate *in vitro* antioxidant capacity (Rastegar et al., 2020). The antioxidant capacity of fruit is associated with its preservation method, storage temperature, antioxidant enzyme activities, and antioxidant accumulation, among other factors (Yu et al., 2021). In the current study, both assays were used to investigate the effects of exogenous MT on the antioxidant capacity of Hami melon fruit during storage (**Figures 4D, E**). DPPH-scavenging capacity for the control and MT groups decrease throughout the storage period, and 0.5 mmol L^–1^ MT treatment markedly inhibits the decrease of the DPPH-scavenging capacity compared with that of the control group.

Consistent with DPPH-scavenging capacity, ABTS-scavenging capacity is also markedly increased for the 0.5 MT group during storage. These results show similar tendencies to those for AsA, TPC, and TFC, and these positive correlations have also been confirmed by previous research (Liu et al., 2018). Furthermore, O_2_^−^ and H_2_O_2_ levels show the opposite trends to those of DPPH- and ABTS-scavenging capacity, and these negative correlations have also been observed by Wang et al. (2021). These results reveal that MT treatment maintain AsA, TPC, and TFC and decrease ROS (O_2_^−^ and H_2_O_2_) in Hami melon fruit, and that this is possibly associated with the enhancement of DPPH- and ABTS-scavenging capacity. Moreover, AsA, TPC, and TFC are related to SOD, POD, CAT, and APX. These results demonstrate that 0.5 mmol L^−1^ MT treatment enhances the antioxidant capacity of Hami melon by activating antioxidant enzymes and antioxidant substances, repressing ROS accumulation and thereby maintaining the postharvest organoleptic quality of Hami melon.

## 5 Conclusions

In summary, different concentrations of MT delay the loss in fruit firmness and weight to varying degrees. The fruit treated with 0.5 mmol L^−1^ MT showed the best effect. Furthermore, melatonin treatment maintains the contents of total soluble solids, titratable acid, ascorbic acid, total phenols, and total flavonoids, reduces the accumulation of ROS and membrane lipid peroxidation, and enhances the activities of antioxidant enzymes in Hami melon fruit. Thus, melatonin is a promising agent for delaying the oxidative senescence of Hami melon fruit and maintaining its postharvest quality, but its sophisticated molecular mechanism needs further study.

## Data availability statement

The original contributions presented in the study are included in the article/supplementary material. Further inquiries can be directed to the corresponding authors.

## Author contributions

YW: Methodology, Software, Writing – original draft. MG: Methodology, Data curation, Formal Analysis, Funding acquisition, Writing – review & editing. WZ: Data curation, Formal Analysis, Writing – review & editing. YG: Resources, Software, Visualization, Writing – review & editing. XM: Investigation, Methodology, Visualization, Writing – review & editing. SC: Conceptualization, Supervision, Writing – review & editing. GC: Conceptualization, Writing – review & editing, Project administration, Funding acquisition.
